# The carbon footprint of beef and beef × dairy crossbred steers under different growing regimens

**DOI:** 10.1093/jas/skaf446

**Published:** 2025-12-18

**Authors:** Matt R Beck, Logan R Thompson, Paul A Beck

**Affiliations:** Department of Animal Science, Texas A&M University, College Station, TX 77845; Department of Animal Science and Industry, Kansas State University, Manhattan, KS 66506; Department of Animal and Food Sciences, Oklahoma State University, Stillwater, OK 74078

**Keywords:** beef production, finishing cattle, grazing cattle, greenhouse gas emissions, partial life cycle assessment

## Abstract

Beef × dairy crossbred steers are becoming an increasingly important source of feeder cattle within the U.S. beef supply chain. However, there has been minimal investigation into greenhouse gas emissions [GHG; kg carbon dioxide equivalence (CO_2_e)] or carbon footprint [kg CO_2_e/kg hot carcass weight (HCW); C footprint] from beef × dairy crossbred steers in the U.S. Furthermore, how beef × dairy crossbred steers compare with beef steers subjected to different growing systems (calf-fed vs. stocker) is not well explored. The objective of this analysis was to compare the C footprint of beef × dairy and beef steers, with or without a stocker phase, from growing through finish. International Panel on Climate Change tier 2 methodology was employed using previously published performance data. Beef and beef × dairy crossbred steers were either grown in a season-long stocker system where they grazed mixed-grass prairie prior to finishing (yearling-fed) or were grown in the feedlot (calf-fed). Cattle grown in a stocker system had 4.6% and 11.3% greater C footprint than calf-fed steers for beef × dairy and beef steers, respectively. This increase was due to greater emissions arising during grazing compared with confined feeding systems. Furthermore, beef × dairy crossbred steers had 11.7% greater C footprint when calf-fed and 3.8% greater C footprint when yearling-fed compared with beef steers. These results suggest that direct emissions arising from growing cattle through finish are greater from beef × dairy sourced steers than beef. However, considering the known importance of dam emissions to overall C footprint, we conducted a secondary analysis to examine how this impacts the final emission estimate. When dam emissions are allocated to these C footprints, beef × dairy crossbred steers will have a considerably lower predicted C footprint compared with beef steers, as only a fraction of dairy dam’s GHG emissions will be allocated to beef production. Future research is needed which expands system boundaries to more aptly compare the C footprint of beef produced from beef and beef × dairy crossbred steers.

## Teaser

Beef × dairy crossbred steers have a greater carbon footprint than beef steers during the stocker and finishing phases. However, beef × dairy steers will have a lower carbon footprint when dam emissions are included in the system boundaries.

## Introduction

Greenhouse gas (GHG) emissions are a side effect of food production, including cattle production, with agriculture, forestry, and other land use accounting for 22% of global anthropogenic GHG emissions (Dhakal et al., 2023). According to the sixth assessment report by the International Panel on Climate Change (IPCC), enteric methane (CH_4_) from ruminant livestock accounts for 5% of global anthropogenic GHG emissions (Dhakal et al., 2023). In the U.S., enteric CH_4_ represented 3% of GHG emissions with 2.2% from the beef industry in 2022 (EPA, 2024). Other sources of GHG emission in beef production arise from manure CH_4_ and nitrous oxide (N_2_O), land N_2_O, enteric N_2_O, anthropogenic carbon dioxide (CO_2_) from fossil fuel and energy use, and upstream sources of CO_2_ equivalence (CO_2_e) which are emissions associated with the production of resources used on cattle operations (Rotz et al., 2019). When considering these GHG sources, Rotz et al. (2019) estimated that U.S. beef production emits 21.3 kg of CO_2_e per kg of hot carcass weight (HCW). These estimates were derived from a full life cycle assessment (LCA), which accounted for all the sources of GHG emissions across all the sectors of the beef supply chain. However, partial LCA have been used in the past when available information is incomplete, or when the desired objectives of the analysis do not necessitate a full LCA (Stackhouse-Lawson et al., 2012; Stanley et al., 2018; Cole et al., 2020; Crawford et al., 2022).

Advancements in the use of sexed semen have resulted in greater use in the dairy industry. Dairy producers can breed their highest producing cows with female sexed semen for replacement heifers and then breed the remaining, less efficient herd mates with semen from beef breeds (Berry, 2021). Dairy derived beef represents a relatively large proportion of the U.S. total beef production, contributing 21% of beef produced in 2019 (Moreira et al., 2021). However, purebred dairy calves typically have lesser growth performance, feed efficiency, and carcass yield compared with beef breeds (Berry, 2021). By producing beef × dairy crossbred cattle, dairy farmers can provide the beef industry with more efficient animals with higher yielding carcasses, which increases the profitability of both industries. Accordingly, beef × dairy crossbred cattle availability, use, and value has been increasing (Basiel and Felix, 2022). The expanded market for beef × dairy crossbred cattle largely explains the 67% increase in U.S. domestic beef semen sales in 2024 relative to 2019, according to the National Association of Animal Breeders (NAAB, 2025). The increased value of the beef × dairy system accordingly represents a win-win for dairy and beef producers. There has been much work assessing the impact of dairy derived beef on the C footprint (kg of CO_2_e per kg of HCW) in Europe (Murphy et al., 2017, 2018; Kearney et al., 2023, 2024) and in the U.S. (Stackhouse-Lawson et al., 2012; Rotz et al., 2019). However, to our knowledge, the C footprint of U.S. beef × dairy crossbred and beef cattle under similar production systems has not been assessed. Furthermore, the C footprint of beef × dairy crossbred steers grown in confinement (calf-fed) or on pasture (stocker) has not been assessed and this comparison has rarely been made for beef steers. Accordingly, the objective of the current research was to estimate the C footprint associated with beef × dairy and beef steers that were either grown in confinement or on pasture using IPCC tier 2 methodology (IPCC, 2019). We hypothesized that beef steers have a lower C footprint when considering emissions arising from the post-weaning growing through slaughter. We further hypothesized that calf-fed steers have a lower C footprint than steers that underwent a stocker phase.

## Materials and Methods

**Figure 1.**
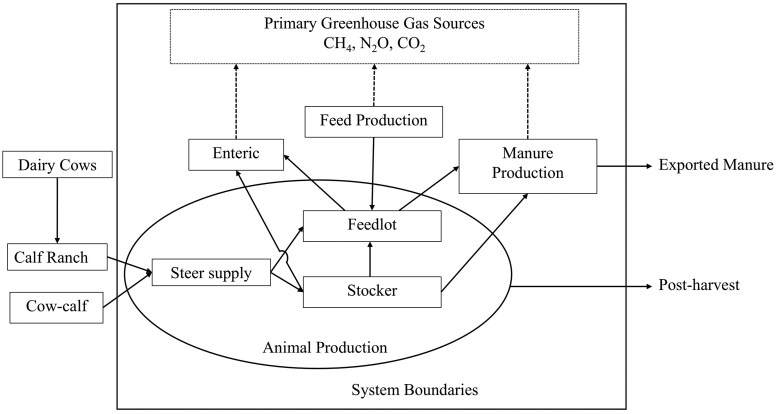
System boundaries (contained within the square) and primary sources of Greenhouse gas emissions that were considered.

A partial LCA was conducted using data from Beck and DeVuyst (2025) that is published in Grote et al. (2025) using IPCC tier 2 methodology (IPCC, 2019). The system boundaries included feed emissions and calf emissions from the growing phase (stocker or backgrounder) through slaughter. Within this manuscript, cattle that entered straight into the feedlot are referred to as calf-fed, while those that underwent a growing phase on pasture are referred to as yearling-fed. All primary GHG generated from enteric fermentation, manure management and feed production were included in this analysis. All gases (CO_2_, N_2_O, CH_4_) were converted to CO_2_e using global warming potential over a 100-yr time horizon. Figure 1 depicts the system boundaries employed and sources of emissions that were considered in the current analysis. Cow emissions were calculated for dairy (Rotz et al., 2021) and beef (Rotz et al., 2019) and emissions from the calf-ranch for the beef × dairy crossbred steers was obtained from Stackhouse-Lawson et al. (2012). These estimates are not reported as results as they are not directly arising from the current analysis, however, they are used to further the discussion. The dataset of Beck and DeVuyst (2025) included data collected from 160 beef and 184 beef × dairy crossbred steers. These cattle were either placed directly into the feedyard (calf-fed; *n* = 85 beef and 109 beef × dairy steers) or grazed on native range for 145 d in southwestern Oklahoma (yearling fed; *n* = 75 beef and 75 beef × dairy steers). Table 1 provides a summary of relevant information and readers are referred to Grote et al. (2025) for a more complete description of animal management.

**Table 1.** Characteristics of the steers used in the current carbon-footprint analysis

	Beef		Beef × dairy	
Item^1^	Calf-fed	Yearling-fed	Calf-fed	Yearling-fed
**Stocker**				
***n***	0	75	0	75
** Days grazed**	0	145	0	145
** Initial BW, kg**	–	267.4 ± 19.73	–	276.0 ± 15.68
** Final BW, kg**	–	379.9 ± 33.23	–	356.0 ± 25.56
** ADG, kg/d**	–	0.8 ± 0.16	–	0.6 ± 0.14
** Death loss, %**	–	2.7	–	2.7
** Average day of death**	–	26.5	–	62.5
**Finisher**				
***n***	85	73	109	73
** Days on feed**	296.3 ± 20.96	200.7 ± 6.84	302.2 ± 12.10	202.4 ± 3.52
** Initial BW, kg**	268.0 ± 34.16	359.4 ± 30.33	261.9 ± 15.37	338.0 ± 23.80
** Final BW, kg**	665.1 ± 59.64	691.3 ± 68.28	709.6 ± 46.33	726.5 ± 51.55
** ADG, kg/d**	1.3 ± 0.19	1.7 ± 0.31	1.5 ± 0.17	1.9 ± 0.26
** DMI, kg/d**	8.4 ± 1.17	9.6 ± 1.58	9.2 ± 0.97	10.9 ± 1.30
** HCW, kg**	429.9 ± 39.03	440.3 ± 44.06	439.2 ± 28.67	442.7 ± 30.53
** Death loss, %**	8.2	2.7	13.8	2.7
** Average day of death**	157.4	95.0	177.6	143

Beef or beef × dairy crossbred steers were either grown in a feedlot (Calf-fed) or on pasture (Yearling) and then finished in a commercial feedlot [Adapted from Grote et al. (2025); available from Beck and DeVuyst (2025)].

1 BW, body weight; ADG, average daily gain; DMI, dry matter intake; HCW, hot carcass weight.

### Enteric CH_4_ emissions

The tier 2 methodology was used to determine enteric CH_4_ emissions from cattle (IPCC, 2019). First, gross energy intake (GEI, MJ/d) was modeled as:


(1) $$\mathrm{GEI}\;=\frac{\left(\frac{{NE}_{m}+\;{NE}_{a}}{\mathrm{REM}}\;+\;\frac{{NE}_{g}}{\mathrm{REG}}\right)}{\left(\mathrm{DER}\right)}$$

Where NEm is MJ of net energy for maintenance required per day (0.322 MJ per kg of average metabolic BW); net energy for activity (NEa) is MJ of NEm needed for activity (17% of NEm required); and net energy for gain (NEg) is MJ of NEg required per day calculated as:


(2) $$\mathrm{NEg}\;(\frac{MJ}{d})=22.02\times {\left(\frac{\mathrm{average}\;BW}{\mathrm{Final}\;BW}\right)}^{0.75}\;\times {\mathrm{ADG}}^{1.0967}$$

REM and REG in equation 1 are the ratios of net energy available for maintenance and growth, respectively, to digestible energy consumed. The REM is calculated according to equation 10.14 and REG by equation 10.15 of IPCC (2019). Finally, DER is the digestible energy-to-gross energy ratio. Once GEI is calculated, enteric CH_4_ (g/d) emissions are calculated as:


(3) $$\mathrm{Enteric}\;{CH}_{4}=\;\frac{\mathrm{GEI}\times Ym}{0.056}$$

Ym is CH_4_ yield expressed as % of GEI, and 0.056 is the amount of MJ per g of CH_4_. The Ym used was 6.3% for the stocker (table 10.12; IPCC, 2019), 4% while fed the backgrounder diet (table 10.12; IPCC, 2019), and 3.24% for the finisher sectors, which is based on a baseline Ym of 3% with a 8% increase adjustment made as the diet contained 1% added fat (Leytem et al., 2024). These emissions were then expressed on a kg per head basis, based on days grazed and on feed. Methane emissions were then expressed as kg of CO_2_e per head by multiplying by 28 (the global warming potential on a 100-yr basis; IPCC, 2021).

### Manure CH_4_ emissions

For both stocker and finishing sectors, volatile solid excretion (kg/hd/d; VS) was calculated as 7.6 kg of VS per 1,000 kg of BW (table 10.13; IPCC, 2019). Manure CH_4_ emissions (kg/head/d) were then calculated as:


(4) $${\mathrm{manure}\;CH}_{4}=VS\;\times B_{0}\times 0.67\times \frac{\mathrm{MCF}}{100}$$

B_0_ is the maximum CH_4_ producing capacity for manure (m^3^ CH_4_ per kg VS), 0.67 is the conversion factor of m^3^ CH_4_ to kg CH_4_, and MCF (%) represents the CH_4_ conversion factor for housing or manure management systems. The B_o_ used was 0.19 for the stocker, the finishing sector (table 10.16A; IPCC, 2019). The MCF was 0.46% for the stocker sector and 4.0% for the finishing sector (table 10.17; IPCC, 2019).

### Manure N_2_O emissions

Direct and indirect N_2_O emissions were determined for the stocker and feedlot sectors. First, total nitrogen excretion (Nex; g/d) was calculated as the sum urine and fecal nitrogen excretion. Urine nitrogen excretion was calculated using equation 16-1 and fecal nitrogen using equation 16-3 of (NASEM, 2016). Nitrogen intake of stocker cattle was calculated by first estimating DMI required for maintenance and gain (NASEM, 2016) and then multiplying this by the N content of the forage. Net energy for maintenance and gain and CP content of the forage was obtained from a feed composition table with feeds typical of the southern plains region (Gross et al., 2025). Direct N_2_O emissions (kg/hd/d) were then calculated as:


(5) $$\mathrm{Direct}\;N_{2}O=(\frac{\mathrm{Nex}}{1,000})\times {EF}_{N_{2}O}\times (\frac{44}{28})$$

Where ${EF}_{N_{2}O}$ is the direct N_2_O emission factor (kg N_2_O-N per kg Nex), which is 0.02 for the finisher sector (table 10.21; IPCC, 2019) and 0.004 for the stocker sector (table 11.1; IPCC, 2019), and 44 divided by 28 converts N_2_O-N to N_2_O emissions. Indirect manure N_2_O emissions from ammonia emissions and nitrate leaching were calculated according to IPCC (2019) recommendations for the finishing sector (Chapter 10) and stocker sector (Chapter 11). Indirect manure N_2_O from housing (dry lot pen surface) and manure management (dry storage) were calculated for the finishing sector. Indirect manure N_2_O from managed soils were considered for the stocker sector as all manure was maintained on pasture.

### Feed emissions

The diets used during finishing are presented by Grote et al. (2025). The initial step-up diets and final finishing diets were total mixed rations containing alfalfa hay as the roughage, steam flaked corn as the primary concentrate energy source, and wet distillers’ grains as the primary protein source. Greenhouse gas emissions of corn grain and alfalfa production were modeled using the Farm Energy Analysis Tool (FEAT) model (Camargo et al., 2013). The FEAT model accounts for emissions arising from transportation of feed from farm, grain drying, on-farm fuel, insecticide, herbicide, lime, potassium, phosphorus, and synthetic nitrogen use. Using this model, corn grain production resulted in 0.31 kg CO_2_e and alfalfa yielded 0.20 kg CO_2_e per kg dry matter (DM). These emissions were based on the USDA-NASS (2024) national yields in 2023 for corn grain (10.85 metric tons/ha) and alfalfa (7.55 metric tons/ha). Emissions to steam-flake the corn were based on 26.3 kL of natural gas and 17.5 kWh of electricity needed to process 1,000 kg of corn DM (Cole et al., 2020). Next, 0.668 kg of CO_2_e were emitted per kL and 0.629 kg CO_2_e were emitted per kWh (Rotz et al., 2019). For wet distillers’ grains the corn grain production GHG emissions were separated between ethanol and the beef industry by economic allocation. For this, 22% of the corn grain production GHG emissions were assigned to the beef industry as it has been shown that 22% of ethanol plant’s revenue stems from the sale of distillers’ grains (Dennis et al., 2024). For the remainder of the ingredients with little known emission intensities (corn syrup, breading, fat, vitamin, and mineral premix), we assigned 0.46 kg CO_2_e/kg of ingredient DM as employed by Cole et al. (2020) and Crawford et al. (2022). Finally, emissions to transport the ingredients to the feedlot were calculated. For the current analysis, each ingredient was transported 200-km, except for wet distillers’ grains which traveled 50-km (Cole et al., 2020; Crawford et al., 2022). The kg of CO_2_e to transport the ingredients to the feedyard was calculated as 146.9 g of CO_2_e were emitted per ton (as-fed basis) of ingredient per km traveled (Hünerberg et al., 2014). The total amount of each ingredient consumed for each breed type and growing system was calculated based on ration composition, average intake of each ration, and the days fed the rations. The total consumption of each ingredient was multiplied by the C footprint for each ingredient (kg CO_2_e per kg of ingredient) to calculate total kg CO_2_e emissions from feed production.

### Death loss emissions

Death loss represents a significant economic loss to producers, but also a source of GHG emissions. There were significant differences in death loss based on cattle type and growing system. Accordingly, these differences in death loss need to be accounted for in the current analysis for both the stocker and finishing sectors. To do this, we took the total sum of emissions from the stocker and feedlot sectors (kg CO_2_e/head/d) and multiplied this by death loss percent and the number of days on pasture or on feed when the death loss occurred. The day on feed or pasture that death loss occurred was not recorded and was therefore approximated based on the average day between the last weigh day that data was available and the weigh day that data was first missing for the dead animal.

## Results

On a daily basis, beef steers emitted 16.4% more enteric CH_4_ than beef × dairy steers during the stocker phase, while beef × dairy steers emitted 7.7% more enteric CH_4_ than beef steers during the finisher sector on average across calf-fed and yearling fed systems (Table 2). Enteric CH_4_ emissions accounted for 87.5% and 86.7% of the total stocker emissions for beef and beef × dairy steers, respectively (Table 3). Furthermore, in the stocker phase of production, beef had 15.3% more emissions than beef × dairy steers. During the finisher phase, beef had 12% lower GHG emissions than beef × dairy steers, on average. Over both the finisher and stocker sectors, beef steers had 8.2% lower GHG emissions on average compared with the beef × dairy steers. Finally, beef steers had 7.0% lower C footprint (kg CO_2_e per kg HCW) than beef × dairy steers, when considering only those steers that went through both the stocker and finisher sectors.

**Table 2.** Methane (CH_4_) and nitrous oxide (N_2_O) emissions (g/d) arising from or beef × dairy crossbred steers backgrounded in the feedlot (calf-fed) or grown on native range pasture (yearling)

	Beef		Beef × dairy	
Item^1^	Calf-fed	Yearling-fed	Calf-fed	Yearling-fed
**Stocker**				
** Enteric CH_4_**	0.0	172.1	0.0	147.9
** Manure CH_4_**	0.0	0.00147	0.0	0.00144
** Manure N_2_O**	0.0	0.0025	0.0	0.0022
**Finisher**				
** Enteric CH_4_**	109.5	116.5	117.1	126.4
** Manure CH_4_**	0.018	0.020	0.019	0.21
** Manure N_2_O**	0.0056	0.0063	0.0060	0.0070

1 CH_4_, methane; N_2_O, nitrous oxide.

**Table 3.** Emissions (kg of carbon dioxide equivalence; CO_2_e) arising from or beef × dairy crossbred steers backgrounded in the feedlot (calf-fed) or grown on native range pasture (yearling)

	Beef		Beef × dairy	
Items^1^	Calf-fed	Yearling-fed	Calf-fed	Yearling-fed
**Stocker**				
** Enteric CH_4_**	0.0	698.8	0.0	600.3
** Manure CH_4_**	0.0	0.0060	0.0	0.0058
** Manure N_2_O**	0.0	95.7	0.0	84.3
** Death loss**	0.0	3.9	0.0	7.9
** Total**	0.0	798.4	0.0	692.5
**Finisher**				
** Enteric CH_4_**	908.7	654.6	990.7	716.4
** Manure CH_4_**	0.15	0.11	0.16	0.12
** Manure N_2_O**	439.5	334.3	482.4	376.8
** Feed emissions**	804.6	648.7	897.8	742.6
** Death loss**	94.2	21.2	191.8	35.5
** Total**	2,247.2	1,659.0	2,562.9	1,871.5
**Grand Total**	2,247.2	2,457.4	2,562.9	2,563.9
**kg CO_2_e/kg FBW**	3.38	3.55	3.61	3.53
**kg CO_2_e/kg HCW**	5.23	5.58	5.84	5.79

1 CH_4_, methane; N_2_O, nitrous oxide; FBW, final body weight; HCW, hot carcass weight.

**Figure 2.**
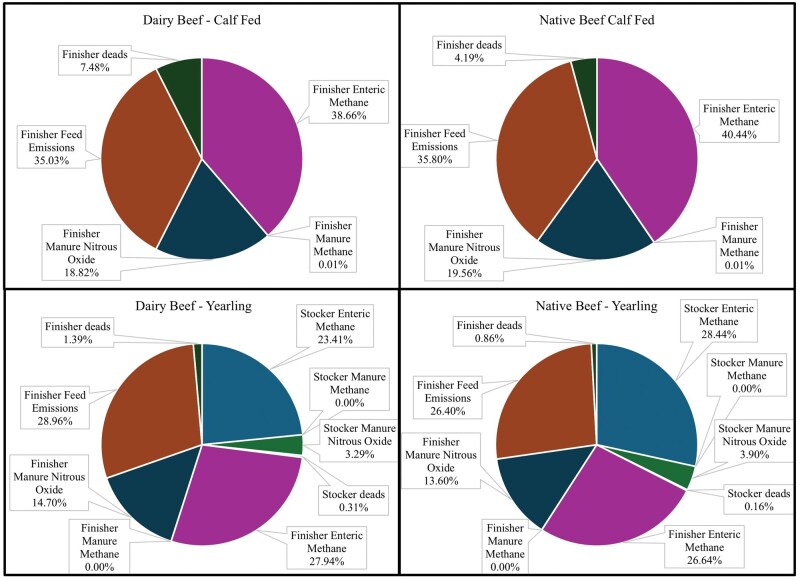
Proportion of total greenhouse gas emissions by source.

The proportion of total GHG emissions produced varied across production systems (Figure 2). For calf-fed steers, finisher enteric CH_4_ emissions represented the largest source of GHG emissions at 38.7% and 40.4%, followed closely by feed emissions at 35.0% and 35.8% for dairy beef and beef steers, respectively. Direct and indirect manure N_2_O emissions represented 18.8% of the calf-fed GHG emissions. Emissions from animals that died in the calf-fed systems accounted for 4.2% and 7.5% for beef and dairy beef steers, respectively.

## Discussion

The current results support the hypothesis, that beef × dairy steers have a greater C footprint compared to beef steers when only GHG emissions from growing through slaughter are considered. However, this initial analysis is an incomplete picture of the emissions generated during the production of finishing steer. Importantly, when dam emissions are allocated to beef production, the beef steers have a greater C footprint relative to the beef × dairy steers considering not all the dairy dam emissions are assigned to the production of that steer. While the information needed to directly model dam emissions was not available in the same granularity of the steer production data, some estimates are made below to further the discussion around this point. Lastly, we further hypothesized that calf-fed steers have a lower C footprint than steers grown on pasture, which the current results support and will be discussed below in more detail.

### Dam emission estimates and allocation

The data necessary to directly estimate dam emissions is unknown from the current set of calves, we recognize that dam emissions will represent a large proportion of the kg of CO_2_e produced per kg of HCW, particularly for beef cattle. For example, Rotz et al. (2019) estimated that the cow-calf sector accounted for approximately 70% of the C footprint of U.S. beef. However, for beef × dairy steers the dam’s importance will be dictated by how dam emissions are allocated, and therefore will have a large impact on the relative change in the C footprint. Based on the LCA conducted by Rotz et al. (2021), the U.S. dairy cow emits 10,588.24 kg CO_2_e per year. The dairy cow obviously produces a calf to commence lactation and produce milk. Accordingly, there have been several proposed methods to allocate the dairy cow’s emissions to beef. These include: 1) no allocation, the full emissions by the dairy cow are attributed to milk production; 2) Cause-effect physical (“biological”) allocation; 3) Economic allocation, the emissions are attributed to milk and beef based on the proportion of revenue to the dairy farm from milk and the sale of the calf; and 4) system expansion (Cederberg and Stadig, 2003). Cederberg and Stadig (2003) recommended the system expansion approach which aims to avoid allocation by expanding the milk system to include the alternative ways of producing the co-products from milk production (i.e., dairy calves and cull cows entering beef production). However, the U.S. dairy industry and beef industry are sufficiently distinct, so that most researchers have opted for other allocation methods.

Several LCA analyses that have included meat production from dairy production systems opted for a biological allocation (Thoma et al., 2013; Rotz et al., 2019, 2021), whereas others have utilized an economic allocation procedure (Rotz et al., 2010; Stackhouse-Lawson et al., 2012). The International Dairy Federation (IDF, 2015) recommends a biological allocation when attributing dairy emissions between meat and milk as it should be relatively consistent in allocation as the biological needs of a lactating dairy cow are inherent to her production potential. In comparison, an economic allocation approach is highly variable year by year and depends on milk price and calf price. Economic allocation also introduces bias to the C footprint of straightbred dairy calves entering the beef supply chain, by allocating less dairy cow emissions to that meat source because straightbred dairy calves are less valuable. For consistency, repeatability across years and dairy calf breed, and to align with current standards, a biological allocation approach was deemed the most appropriate for the purpose of our analysis. Additionally, 326 kg of CO_2_e was added to the beef × dairy crossbred steers to account for emissions while at the calf-ranch (Stackhouse-Lawson et al., 2012).

According to Rotz et al. (2019), the U.S. beef cow produces 5,464.6 kg of CO_2_e per year. These emissions are attributable to the beef industry and increase the C footprint of our estimates to 17.94 and 17.99 kg CO_2_e per kg HCW for calf-fed and yearling fed beef, respectively (Table 4). Using the estimated biological allocation rate of 9.4% employed by Thoma et al. (2013) approximately 995.3 kg CO_2_e are allocated to the beef × dairy crossbred steers from their dams and 326 kg CO_2_e from the calf ranch (Stackhouse-Lawson et al., 2012). This increases the predicted C footprint of beef × dairy emissions to 8.84 and 8.78 for calf-fed and yearling-fed steers, respectively. Accordingly, based on these estimates, beef × dairy steers have 51% lower C footprint emissions relative to beef steers. This difference is due to the much lower dam emissions that were allocated. These comparisons are rough estimates based on emission estimates from previous analyses. A more complete LCA is needed to confirm the relationships these estimates have demonstrated.

**Table 4.** Estimated cow emissions [kg of carbon dioxide equivalence (CO_2_e)], total estimated emissions and the carbon footprint from or beef × dairy crossbred steers that were backgrounded in the feedlot (calf-fed) or grown on native range pasture (yearling)

	Beef		Beef × dairy	
Item^1^	Calf-fed	Yearling-fed	Calf-fed	Yearling-fed
**Cow emissions^2^**	5,464.6	5,464.6	10,588.2	10,588.2
**Cow emissions allocated to beef**	5,464.6	5,464.6	995.3	995.3
**Calf ranch emissions^3^**	–	–	326.0	326.0
**kg CO_2_e emissions^4^**	7,711.8	7,922.0	3,884.2	3,885.2
**kg CO_2_e/kg HCW**	17.94	17.99	8.84	8.78

1 HCW, hot carcass weight.

2 From Rotz et al. (2019).

3 From Stackhouse-Lawson et al. (2012).

4 Emission from stocker and finisher sectors plus the cow emissions that were allocated to beef.

### Stocker sector

Estimated enteric CH_4_ emissions were 172 g/d for beef steers during the stocker sector. The beef steers daily CH_4_ emissions are like CH_4_ emissions measured from cattle grazing tallgrass prairie pastures in Oklahoma (188.5 g/d; Beck et al., 2019) and shortgrass prairie in northern Colorado (185.8 g/d; Raynor et al., 2024) using the GreenFeed systems (C-Lock Inc., Rapid City, SD; Gunter and Beck, 2018). These similarities suggest that our estimated CH_4_ emission rates align well with previous emission rates that were measured directly.

Excluding emissions from death loss, beef steers emitted more GHG from all estimated sources and had 16% greater emissions than beef × dairy steers in the stocker phase of production. The beef steers had faster rates of gain (33.3% high average daily gain; Table 1) and subsequently greater estimated gross energy intake. Accordingly, the greater estimated gross energy intake yielded greater CH_4_ emission estimates. Some of the lower average daily gain and presumably lower dry matter intake may be due to dietary neophobia and beef × dairy steers lacking grazing experience (Beck and Gregorini, 2021). Raynor et al. (2024) conducted a grazing experiment in northern Colorado with sourced local cattle (experienced) and cattle that originated from south-central Nebraska (naïve). Like the current analysis, the experienced cattle had much greater ADG and 20% greater CH_4_ emissions than the naïve cattle. Another potential explanation for the lower average daily gain by the beef × dairy steers during the stocker phase may also be due to differences in maintenance energy requirements. Dairy breeds have 20% greater maintenance energy requirements relative to Bos taurus breeds (NASEM, 2016). Accordingly, the beef × dairy steers likely had greater maintenance energy requirement than the beef steers; however, how much greater is largely unknown.

### Finisher sector

The yearling fed steers emitted 116.5 to 126.4 g of enteric CH_4_ per head per day during the finishing phase. These enteric CH_4_ emissions fall within the range previously reported for beef cattle fed finishing diets (Proctor et al., 2024; Galyean et al., 2025). The calf-fed steers had lower daily CH_4_ emission rates than the yearling-steers, which makes intuitive sense as they had lighter average body weights and DM intake (DMI) while on feed. The calf-fed steers emitted 2,405 kg CO_2_e and the yearling fed steers emitted 1,765 kg CO_2_e, on average in the finisher sector. These estimates are greater than those reported by Cole et al. (2020) and Crawford et al. (2022), with GHG emission estimates ranging from 1,188 to 1,397.0 and 1,317.81 to 1,445.55, respectively. These differences are largely driven by days on feed. In the current analysis, cattle were fed for an average of 299.2 and 201.6 d for calf-fed and yearling fed steers, respectively, while cattle were on feed for 161 d and 182 d for Cole et al. (2020) and Crawford et al. (2022), respectively. If we express the range in CO_2_e emissions from the current study on a daily basis (8.04 to 8.76), they align closely with the previous analyses (7.24 to 8.68 kg CO_2_e per day; Cole et al., 2020; Crawford et al., 2022).

Some of the discrepancy observed between the current analysis, and the previously published partial LCA (Cole et al., 2020; Crawford et al., 2022) arise from our accounting for death loss. When only finisher emissions are considered, the C footprint estimate for yearling-fed beef steers is 3.77. This estimate is 5.8% higher than the average estimate of Cole et al. (2020) and 9.2% higher than Crawford et al. (2022); however, our calculations estimated GHG emissions due to dead animals. During the finishing phase, death loss accounted for 0.9% to 7.5% of total GHG emissions (Figure 2). Ignoring death loss and using only the finishing phase GHG emissions from yearling fed cattle yields an estimated C footprint of 3.72 kg CO_2_e/kg HCW from the finishing period only, which is 5.4% greater than Cole et al. (2020) and 7.8% greater than Crawford et al. (2022).

### Calf-fed vs yearling-fed

On average, calf-fed steers had a 2.6% lower C footprint (kg CO_2_e/kg of HCW) than yearling-fed steers grown on pasture when ignoring dam emissions. This difference was greater for beef than beef × dairy steers. The calf-fed beef steers had 6.3% lower C footprint than the beef steers that were grown on pasture, while the calf-fed and yearling-fed beef × dairy steers were similar (<1% difference). When estimated emissions from the cow-calf sector were included, calf-fed beef steers had 2.7% lower GHG emissions than beef steers grown on pasture. This estimate aligns closely with Johnson et al. (2003), who reported that cattle who undergo a stocker phase have 4% greater GHG emissions than calf-feds. Furthermore, Stackhouse-Lawson et al. (2012) reported that including a stocker phase in California beef production adds 6% more GHG emissions than a system without a stocker phase.

One might see these results and suggest the removal of the U.S. stocker industry to reduce the C footprint of beef production. However, despite having larger GHG emissions and C footprint compared with a calf-fed system, the stocker system provides an important benefit to the beef industry. Peel (2003) reviewed the U.S. stocker industry and summarized its benefit into three economic roles. The first is a production role where cattle are grown and the quality and uniformity of feeder cattle are improved. The second economic role of the stocker industry is an inventory role. Approximately, 55.6% of beef calves are born during the months of February, March, and April (USDA-NAHMS, 2020). By entering a wide range of stocker systems for varying lengths of time, the stocker industry spreads out the supply of feeder cattle that are available to enter the feedlot (Peel, 2003). Finally, the stocker industry provides a market balancing role. The stocker industry is inherently flexible and can adjust based on stocker cattle availability and price, which is dependent on cattle inventory, cattle price, forage availability, and grain price (Peel, 2003). The market balancing role of the stocker sector can most clearly be seen in the close positive relationship between feedlot cost of gains and stocker value of gains (Beck et al., 2013). This relationship indicates feeders are incentivizing the stocker sector to grow animals when the feedlot cost of gain is high. The differences in the C footprint associated with beef derived from calf-fed and stocker systems is important to note; however, consideration of the other services that the stocker industry provides must be considered when assessing the overall sustainability of the stocker industry.

## Conclusions

The current partial LCA provides several significant insights. Beef × dairy crossbred steers have greater emissions than beef steers within the stocker and finisher phases of production. Next, yearling fed steers that underwent a stocker phase had greater emissions than calf-fed steers. Finally, beef × dairy steers will have lower predicted C footprints than beef steers when dam emissions are attributed; however, how much lower the C footprints are will largely depend on how the dam emissions are allocated. Future research should expand system boundaries to more precisely estimate dam emissions to fully account for the C footprint of beef × dairy steers.

## Abbreviations:

ADG: average daily gain
C footprint: carbon footprint
CH_4_: methane
CO_2_: carbon dioxide
CO_2_e: carbon dioxide equivalence
DER: digestible energy-to-gross energy ratio
DM: dry matter
${EF}_{N_{2}O}$: direct nitrous oxide emission factor
DMI: dry matter intake
FEAT: Farm Energy Analysis Tool
GEI: gross energy intake
GHG: Greenhouse gas emissions
HCW: hot carcass weight
IPCC: International Panel on Climate Change
LCA: life cycle assessment
N_2_O: nitrous oxide
NEa: net energy for activity
NEg: net energy for gain
NEm: net energy for maintenance
Nex: total nitrogen excretion
REG: ratio of net energy available for gain
REM: ratio of net energy available for maintenance
VS: volatile solids
Ym: methane yield

## References

[skaf446-B1] Basiel B. L. , Felix T. L. 2022. Board invited review: crossbreeding beef × dairy cattle for the modern beef production system. Transl. Anim. Sci. 6(2):txac025. 10.1093/tas/txac025 35399737 PMC8989152

[skaf446-B2] Beck M. R. , Gregorini P. 2021. Animal design through functional dietary diversity for future productive landscapes. Front. Sustain. Food Syst. 5:546581. 10.3389/fsufs.2021.546581

[skaf446-B3] Beck P. A , DeVuyst E. 2025. Impact of management of composite dairy × beef crossbreds on economic and environmental sustainability of beef production. Mendeley Data. V2. 10.17632/rn6g436yx2.2

[skaf446-B4] Beck M. R. , Thompson L. R., Williams G. D., Place S. E., Gunter S. A., Reuter R. R. 2019. Fat supplements differing in physical form improve performance but divergently influence methane emissions of grazing beef cattle. Anim. Feed Sci. Technol. 254:114210. 10.1016/j.anifeedsci.2019.114210

[skaf446-B5] Beck P. A. , Anders M., Watkins B., Gunter S. A., Hubbell D., Gadberry M. S. 2013. 2011 and 2012 early careers achievement awards: improving the production, environmental, and economic efficiency of the stocker cattle industry in the southeastern United States. J. Anim. Sci. 91(6):2456–2466. 10.2527/jas.2012-5873 23243161

[skaf446-B6] Berry D. P. 2021. Invited review: beef-on-dairy—the generation of crossbred beef × dairy cattle. J. Dairy Sci. 104(4):3789–3819. 10.3168/jds.2020-19519 33663845

[skaf446-B7] Camargo G. G. T. , Ryan M. R., Richard T. L. 2013. Energy use and greenhouse gas emissions from crop production using the farm energy analysis tool. Bioscience. 63(4):263–273. 10.1525/bio.2013.63.4.6

[skaf446-B8] Cederberg C. , Stadig M. 2003. System expansion and allocation in life cycle assessment of milk and beef production. Int. J. LCA. 8(6):350–356. 10.1065/lca2003.07.126

[skaf446-B9] Cole N. A. , Parker D. B., Brown M. S., Jennings J. S., Hales K. E., Gunter S. A. 2020. Effects of steam flaking on the carbon footprint of finishing beef cattle. Transl. Anim. Sci. 4(Suppl 1):S84–S89. 10.1093/tas/txaa110 33381726 PMC7754218

[skaf446-B10] Crawford D. M. , Hales K. E., Smock T. M., Cole N. A., Samuelson K. L. 2022. Effects of changes in finishing diets and growth technologies on animal growth performance and the carbon footprint of cattle feeding: 1990 to 2020. Appl. Anim. Sci. 38(1):47–61. 10.15232/aas.2021-02199

[skaf446-B11] Dennis E. , Gertner D., Erickson G. 2024. Economic research on ethanol feed-use coproducts: a review, synthesis, and path forward. Animals. 14(11):1551. 10.3390/ani14111551 PMC11171241 38891596

[skaf446-B12] Dhakal, S. , Minx J. C., Toth F. L., Abdel-Aziz A., Figueroa Meza M. J., Hubacek K., Jonckheer I. G. C., Kim Y., Nemet G. G., Pachauri S., Tan X. C., Wiedmann T. 2023. Emissions trends and drivers. In: P. R. Shukla, Skea J., Slade R., Al Khourdajie A., van Diemen R., McCollum D., Pathak M., Some S., Vyas P., Fradera R., Belkacemi M., Hasifa A., Lisboa G., Luz S., Malley J., editors. Climate change 2022: mitigation of climate change. Contribution of working group III to the sixth assessment report of the intergovernmental panel on climate change. Cambridge, UK: Cambridge University Press; p. 215–294.

[skaf446-B13] EPA. 2024. Inventory of U.S. Greenhouse gas emissions and sinks: 1990-2022. Washington (DC): U.S. Environmental Protection Agency.

[skaf446-B14] Galyean M. L. , Hales K. E., Holland B. P. 2025. Predicting methane emissions from feedlot cattle and application of prediction equations to a synthetic feedlot steer population. Appl. Anim. Sci. 41(2):119–128. 10.15232/aas.2024-02664

[skaf446-B15] Gunter S. A. , Beck M. R. 2018. Measuring the respiratory gas exchange by grazing cattle using an automated, open-circuit gas quantification system. Transl. Anim. Sci. 2(1):11–18. 10.1093/tas/txx009 32704685 PMC7200863

[skaf446-B16] Gross , Lalman M. A., D. L., Beck P. A. 2025. Cowculator. Available from: https://extension.okstate.edu/programs/cowculator/ (Accessed 1 May 2025).

[skaf446-B17] Grote A. , Fanning T., DeVuyst E., Grigsby Z., Crosswhite J., Beck P. 2025. A commercial scale evaluation of the effects of post-weaning management system of dairy × beef hybrid steers compared to native beef steers on performance, carcass characteristics and net returns. Transl. Anim. Sci. 9:txaf118. 10.1093/tas/txaf118 40980501 PMC12448398

[skaf446-B18] Hünerberg M. , Little S. M., Beauchemin K. A., McGinn S. M., O’Connor D., Okine E. K., Harstad O. M., Kröbel R., McAllister T. A. 2014. Feeding high concentrations of corn dried distillers’ grains decreases methane, but increases nitrous oxide emissions from beef cattle production. Agric. Syst. 127:19–27. 10.1016/j.agsy.2014.01.005

[skaf446-B19] IDF. 2015. A common carbon footprint approach for the dairy sector—the IDF guide to standard life cycle assessment methodology. In: van Belzen, N, editor. Bulletin 445/2010. International Dairy Federation. Brussels, Belgium. https://www.fil-idf.org/wp-content/uploads/2016/09/Bulletin479-2015_A-common-carbon-footprint-approach-for-the-dairy-sector.CAT.pdf

[skaf446-B20] IPCC. 2019. 2019 Refinement to the 2006 IPCC guidelines for national greenhouse gas inventories. Intergovernmental Panel on Climate Change. Buendia, C., K. Tanabe, A. Kranjc, J., , Baasansuren , M. Fukuda, S. Ngarize, A. Osako, Y. Pyrozhenko, P. Shermanau, and S. Federici, editors. Geneva, Switzerland.

[skaf446-B21] IPCC. 2021. Climate change 2021: the physical science basis. Contribution of working group 1 to the sixth assessment report of the intergovernmental panel on climate change. In: V. Masson-Delmotte, Zhai P., Pirani A., Connors S. L., Pean C., Berger S., Caud N., Chen Y., Goldfarb L., Gomis M. I., Huang M., Leitzell K., Lonnoy E., Matthews J. B. R., Maycock T. K., Waterfield T., Yelekci O., Yu R., Zhou B., editors. Cambridge, UK; New York (NY): Cambridge University Press; p. 2391. 10.1017/9781009157896

[skaf446-B22] Johnson, D. E. , Phetteplace H. W., Seidl A. F., Schneider U. A., McCarl B. A. 2003. Management variations for us beef production systems: effects of greenhouse gas emissions and profitability. Proceedings of the 3rd int. Methane and nitrous oxide mitigation conference. Beijing, China. p. 953–961.

[skaf446-B23] Kearney M. , O’Riordan E. G., Byrne N., Breen J., Crosson P. 2023. Mitigation of greenhouse gas emissions in pasture-based dairy-beef production systems. Agric. Syst. 211:103748. 10.1016/j.agsy.2023.103748

[skaf446-B24] Kearney M. , O’Riordan E. G., McGee M., Breen J., Dunne R., French P., Crosson P. 2024. Bioeconomic and sustainability performance of dairy-beef steer and heifer production systems differing in stocking rate. Livest. Sci. 287:105531. 10.1016/j.livsci.2024.105531

[skaf446-B25] Leytem A. , Archibeque B., S., Cole N. A., Gunter S., Hristov A., Johnson K., Kebreab E., Kohn R., Liao W., Toureene C., Tricarico J. 2024. Chapter 4: quantifying greenhouse gas sources and sinks in animal production systems. In: W. L. Hanson, Itle C., Edquist K., editors. Quantifying greenhouse gas fluxes in agriculture and forestry: methods for entity-scale inventory. Technical bulletin number 1939. 2nd ed. Washington (DC): U.S. Department of Agriculture, Office of the Chief Economist.

[skaf446-B26] Murphy B. , Crosson P., Kelly A. K., Prendiville R. 2017. An economic and greenhouse gas emissions evaluation of pasture-based dairy calf-to-beef production systems. Agric. Syst. 154:124–132. 10.1016/j.agsy.2017.03.007

[skaf446-B27] Murphy B. , Crosson P., Kelly A. K., Prendiville R. 2018. Performance, profitability and greenhouse gas emissions of alternative finishing strategies for Holstein-Friesian bulls and steers. Animal. 12(11):2391–2400. 10.1017/s1751731118000034 29402341

[skaf446-B28] Moreira L. C. , Rosa G. J., Schaefer D. M. 2021. Beef production from cull dairy cows: a review from culling to consumption. J. Anim. Sci. 99(7):1–18. 10.1093/jas/skab192 PMC8281100 34125214

[skaf446-B29] NASEM. 2016. Nutrient requirements of beef cattle. 8th ed. In: M. L. Galyean, Beauchemin K. A., Caton J. S., Cole N. A., Eisemann J. H., Engle T., Erickson G. E., Krehbiel C. R., Lemenager R. P., Tedeschi L. O., editors. Washington (DC): National Academies of Sciences.

[skaf446-B30] National Association of Animal Breeders (NAAB). 2025. Annual reports of semen sales and custom freezing. [accessed September 2, 2025]. Available from: https://www.naab-css.org/semen-sales

[skaf446-B31] Peel D. S. 2003. Beef cattle growing and backgrounding programs. Vet. Clin. North Am. Food Anim. Pract. 19(2):365–385, vi. 10.1016/S0749-0720(03)00032-X 12951738

[skaf446-B32] Proctor J. A. , Smith J. K., Long N. S., Gunter S. A., Gouvêa V. N., Beck M. R. 2024. Utilizing gas flux from automated head chamber systems to estimate dietary energy values for beef cattle fed a finishing diet. J. Anim. Sci. 102:skae167. 10.1093/jas/skae167 38864567 PMC11221065

[skaf446-B33] Raynor E. J. , Schilling-Hazlett A., Place S. E., Martinez J. V., Thompson L. R., Johnston M. K., Jorns T. R., Beck M. R., Kuehn L. A., Derner J. D. et al. 2024. Snapshot of enteric methane emissions from stocker cattle grazing extensive semiarid rangelands. Rangel. Ecol. Manag. 93:77–80. 10.1016/j.rama.2024.01.001

[skaf446-B34] Rotz C. A. , Asem-Hiablie S., Place S., Thoma G. 2019. Environmental footprints of beef cattle production in the United States. Agric. Syst. 169:1–13. 10.1016/j.agsy.2018.11.005

[skaf446-B35] Rotz C. A. , Montes F., Chianese D. S. 2010. The carbon footprint of dairy production systems through partial life cycle assessment. J. Dairy Sci. 93(3):1266–1282. 10.3168/jds.2009-2162 20172247

[skaf446-B36] Rotz C. A. , Stout R., Leytem A., Feyereisen G., Waldrip H., Thoma G., Holly M., Bjorneberg D., Baker J., Vadas P. et al. 2021. Environmental assessment of United States dairy farms. J. Clean Prod. 315:128153. 10.1016/j.jclepro.2021.128153

[skaf446-B38] Stackhouse-Lawson K. R. , Rotz C. A., Oltjen J. W., Mitloehner F. M. 2012. Carbon footprint and ammonia emissions of California beef production systems. J. Anim. Sci. 90(12):4641–4655. 10.2527/jas2011-4653 22952361

[skaf446-B39] Stanley P. L. , Rowntree J. E., Beede D. K., DeLonge M. S., Hamm M. W. 2018. Impacts of soil carbon sequestration on life cycle greenhouse gas emissions in Midwestern USA beef finishing systems. Agric. Syst. 162:249–258. 10.1016/j.agsy.2018.02.003

[skaf446-B40] Thoma G. , Jolliet O., Wang Y. 2013. A biophysical approach to allocation of life cycle environmental burdens for fluid milk supply chain analysis. Int. Dairy J. 31:S41–S49. 10.1016/j.idairyj.2012.08.012

[skaf446-B41] USDA-NAHMS. 2020. Beef cow-calf management practices in the United States. United States Department of Agriculture, Fort Collins (CO). Available from: https://www.aphis.usda.gov/sites/default/files/beef2017_dr_parti.pdf (Accessed 1 May 2025).

[skaf446-B42] USDA-NASS. 2024. Press release. Washington (DC). Available from: https://www.nass.usda.gov/Statistics_by_State/Idaho/Publications/Crops_Press_Releases/2024/CP10.pdf (Accessed 1 May 2025).

